# Association of rs6982567 near *GDF6* with neovascular age-related macular degeneration and polypoidal choroidal vasculopathy in a Han Chinese cohort

**DOI:** 10.1186/1471-2415-14-140

**Published:** 2014-11-22

**Authors:** Yuying Ji, Xiongze Zhang, Kunfang Wu, Yu Su, Meng Li, Chengguo Zuo, Feng Wen

**Affiliations:** State Key Laboratory of Ophthalmology, Zhongshan Ophthalmic Center, Sun Yat-sen University, 54 South Xianlie Road, Guangzhou, 510060 China

**Keywords:** Han Chinese population, Neovascular age-related macular degeneration, Polypoidal choroidal vasculopathy, Polymorphisms, Growth differentiation factor 6

## Abstract

**Background:**

*Growth differentiation factor 6 (GDF6)* has been reported to be a novel disease gene for age-related macular degeneration (AMD) in Caucasians. This study aimed to investigate whether rs6982567 was associated with neovascular AMD (nAMD) or polypoidal choroidal vasculopathy (PCV) in a Han Chinese cohort.

**Methods:**

A total of 612 participants (251 PCV patients, 157 nAMD patients and 204 controls) were included in this study. The SNaPshot system was used to genotype the rs6982567. PLINK software was used to evaluate the genotypes and allele frequencies of patients and controls.

**Results:**

The allele frequencies of rs6982567 were not significantly associated with nAMD, PCV or PCV and nAMD combined. Subjects with the TT genotype had a 2.42-fold greater risk of PCV (95% confidence interval, 1.07-5.43, p = 0.0290) than subjects with CC genotype. A recessive model of rs6982567 was statistically significantly associated with PCV (odds ratio, 2.29; 95% confidence interval, 1.04-5.05; p = 0.0351). However, the association did not withstand stringent Bonferroni correction. There were no significant differences in genotype distributions or models in nAMD.

**Conclusions:**

There was a possible weak association between the rs6982567 near *GDF6* and PCV in this replication study with an independent Han Chinese cohort. A complete survey of the *GDF6* locus with a larger sample size is needed in future studies.

## Background

Age-related macular degeneration (AMD) is a major cause of irreversible blindness around the world [1]. Neovascular AMD (nAMD) is one process of advanced AMD, characterised by typical choroidal neovascularisation (CNV) that can cause severe vision loss [2]. Polypoidal choroidal vasculopathy (PCV) is an exudative maculopathy marked by branching networks of inner choroidal vessels and a nodular appearance on stereoscopic view with indocyanine green angiography (ICGA) [3]. Emerging evidence has shown that PCV is more common in Asians than in Caucasians [4, 5], and it has been recognised as a special type of CNV [6, 7]. However, the prevalence, clinical features and treatment guidelines are somewhat different. Although nAMD and PCV share some common genetic determinants, such as the *complement factor H* gene *(CFH)* and the *high temperature requirement factor A1 (HTRA1)* gene [8, 9], they are not the same. For example, *superkiller viralicidic activity 2-like (SKIV2 L),* in the entire *C2 (complement component 2)-CFB (complement factor B)-RDBP (RD RNA-binding protein)-SKIV2 L* region, is associated with nAMD but not with PCV [10]. The rs5882 near *cholesteryl ester transfer protein (CETP)* is significantly associated with PCV but not with nAMD [11].

*Growth differentiation factor 6 (GDF6)* is a member of transforming growth factor-β (TGF-β) superfamily. It can control eye development by regulating neural and vascular development [12, 13] and can regulate ectoderm patterning [14]. It also plays a role in tissue modelling and remodelling [15]. Recently, a study in a Caucasian cohort identified that a single nucleotide polymorphism (SNP) rs6982567 near *GDF6* was associated with AMD [16]. Given that early AMD, advanced AMD, and PCV were not distinguished in this study, and no research about this SNP has been undertaken in Chinese populations, we conducted this study to determine whether rs6982567 was associated with nAMD and PCV in a Han Chinese population.

## Methods

The study followed the tenets of the Declaration of Helsinki, and the protocol was approved by the institutional review board at the Zhongshan Ophthalmic Center of Sun Yat-sen University (lot number: KYNL015). All of the participants signed written informed consent forms after explanation of the purpose and process of the study.

### Study participants

All of the participants were enrolled from Zhongshan Ophthalmic Center from November 2008 to July 2011. They were all Chinese and were not related to each other. All of the PCV and nAMD patients were newly diagnosed and received no prior treatment. They all underwent bilateral ocular examinations, including best-corrected visual acuity, slit-lamp biomicroscopy, direct ophthalmoscopy, colour fundus photographs, fundus fluorescein angiography (FFA), and ICGA. Diagnosis was made according to the worse eye. PCV was defined as characteristic polypoidal choroidal vascular dilations with branching inner choroidal vascular networks within the first 5 min after indocyanine green injection on ICGA. Typical CNV, observed on both FFA and ICGA, was diagnosed as nAMD. The exclusion criteria included combination with any retinal or choroidal diseases in one or both eyes or other neovascularised maculopathies, such as pathologic myopia, angioid streaks, idiopathic CNV, multifocal choroiditis, et al.

The control subjects were unrelated people aged ≥ 50 years old without family histories of AMD. They all underwent ocular examinations, including visual acuity, slit-lamp biomicroscopy, direct ophthalmoscopy and 50 degree fundus photography. Cases with any macular degeneration or changes, such as drusen, pigment abnormalities, or non-clear media preventing macular visualization, were excluded from the study.

### SNP genotyping

Antecubital vein peripheral blood samples were obtained from all of the participants and were placed in tubes containing ethylene diamine tetraacetic acid. As previously described [17], genomic DNA was isolated from peripheral blood samples using the Nucleospin_Blood XL kit (Machery-Nagal GmbH&Co., KG Düren, Germany), and it was stored at −20°C. Rs6982567 variants were genotyped using the SNaPshot System with an ABI 3730XL genetic analyser (Applied Biosystems, Foster City, CA, USA). The primer sequences were 5′- CATGAAATATCATCCACAGACAA-3′ (forward) and 5′- TCATTCAGTATATGCTCCTTAG-3′ (reverse), and the extension primer was TTTTTTTTTTTTTTGATTTTTTCCACATGTAGTCCT. Genemapper software, version 4.1(Applied Biosystems, Foster City, CA, USA), was used to determine the genotypes of the SNP.

### Statistics

Statistical analysis was performed using SPSS software, version 13.0 (SPSS Inc., Chicago, IL, USA). Student’s unpaired t-test and the chi-square test were used to assess the differences in mean age and sex between the subjects and controls. A p value < 0.05 was considered statistically significant. The exact test included in the PLINK software package, version 1.07, was used to test the deviations from Hardy-Weinberg equilibrium [18]. The chi-square test in PLINK was used to determine the allele frequencies of the SNPs in the case subjects. To assume the genotypic additive model, dominant and recessive model, the logistic option and model option in PLINK were used, respectively. Bonferroni’s method was performed to correct the p values in multiple comparisons, with a p value <0.0056 (0.05÷3÷3) considered to be statistically significant. G*Power software, version 3.1.10, was used to evaluate the post-hoc power [19].

## Results

A total of 612 participants were enrolled: 251 PCV patients, 157 nAMD patients and 204 control individuals. Table 1 showed the clinical baseline characteristics. The mean age of the PCV group (65 ± 8.61 years) was younger than the control group, and the difference was significant (p < 0.001). There were no significant differences in other characteristics, such as sex among the three groups.

**Table 1:** **The clinical characteristics of the participants**

Characteristics	PCV	nAMD	Control	p value*	p value ^†^
Number (n)	251	157	204		
Gender (female/male)	84/167	56/101	80/124	0.204	0.488
Mean age ± SD years	65 ± 8.61	67 ± 9.21	69 ± 9.00	<0.001	0.097
Age ranges (years)	42-85	46-84	50-87		

PCV: polypoidal choroidal vasculopathy.

nAMD: neovascular age-related macular degeneration.

*: PCV group compared with control group.

^†^: nAMD group compared with control group.

For the quality assurance of our samples, the genotyping call rates were 100% for rs6958967 in both the patient and control groups. The genotypes were determined using the SNaPshot method successfully in all of the subjects. To confirm the accuracy of the SNaPshot method, randomly selected subjects (10% of all samples) were analysed by direct sequencing. The consensus rate in the replicate samples (n = 61) was 100%. The results of Hardy-Weinberg equilibrium (HWE) analysis showed no significant deviation in rs6982567, as shown in Table 2. The allele frequencies of rs6982567 were not significantly associated with nAMD, PCV or PCV and nAMD combined. The frequencies of the T allele were 29.12% for PCV, 25.48% for nAMD, and 27.94% for PCV and nAMD combined. The odds ratio (OR) for the risk allele T of rs6982567 was 1.29 for PCV [95% confidence interval (CI), 0.97-1.73, p = 0.0832], 0.91 for nAMD (95% CI, 0.65-1.28, p = 0.6029), and 1.14 for PCV and nAMD combined (95% CI, 0.87-1.49, p = 0.3444).

**Table 2:** **The association test for the minor allele frequency of rs6982567**

Status	HWE	MAF	OR (95% CI)	p value
Control	0.1016	0.2623		
PCV	0.2555	0.2912	1.29(0.97-1.73)	0.0832
nAMD	0.5798	0.2548	0.91(0.65-1.28)	0.6029
PCV + nAMD	0.6167	0.2794	1.14(0.87-1.49)	0.3444

SNP: single nucleotide polymorphism.

PCV: polypoidal choroidal vasculopathy.

nAMD: neovascular age-related macular degeneration.

MAF: minor allele frequency.

HWE: p value of Hardy-Weinberg equilibrium test.

OR: odds ratio.

95% CI: 95% confidence interval.

Minor allele frequencies were calculated based on all study participants.

The genotype association test of rs6982567 was summarised in Table 3. Subjects with the TT genotype had a 2.42-fold times greater risk of PCV (95% CI, 1.07-5.43, p = 0.0290) than subjects with the CC genotype. The variant was significantly associated with PCV under a recessive model (OR, 2.29; 95% CI, 1.04-5.05; p = 0.0351). However, after Bonferroni correction, the p value was insignificant (p > 0.0056). The genotype distributions and additive, dominant and recessive models were not significantly associated with nAMD (all p values >0.05). Combining PCV with nAMD as a group, the genotype distributions and additive and dominant models also showed no significant differences (all p values >0.05). Although there was a marginal association under a recessive model in the PCV and nAMD combined group (p = 0.0487), it could not withstand the Bonferroni correction (p > 0.0056).

**Table 3:** **The association test for the genotype of rs6982567**

		Genotype distribution			Genotype	
**Group**	**Genotype**	**Case**	**Control**	**OR (95%CI) p value**	**Model**	**OR (95%CI) p value**
PCV	CC	117	106	reference	Additive	1.31(0.97-1.78) 0.0747
	TC	110	89	1.12(0.76-1.64) 0.5657	Dominant	1.24(0.86-1.79) 0.2560
	TT	24	9	**2.42(1.07-5.43) 0.0290**	Recessive	**2.29(1.04-5.05) 0.0351**
nAMD	CC	92	106	reference	Additive	0.91(0.65-1.29) 0.5961
	TC	53	89	0.69(0.44-1.07) 0.0931	Dominant	0.76(0.50-1.16) 0.2088
	TT	12	9	1.54(0.62-3.81) 0.3510	Recessive	1.79(0.74-4.37) 0.1936
PCV + nAMD	CC	209	106	reference	Additive	1.14(0.87-1.50)0.3390
	TC	163	89	0.93(0.66-1.32) 0.6801	Dominant	1.03(0.74-1.44) 0.8625
	TT	36	9	2.03(0.94-4.37) 0.0660	Recessive	**2.10(0.99-4.44) 0.0487**

SNP: single nucleotide polymorphism.

PCV: polypoidal choroidal vasculopathy.

nAMD: neovascular age-related macular degeneration.

OR: odds ratio; 95% CI: 95% confidence interval.

The present sample size showed >80% power to detect significant association for power calculations (α < 0.017), and the effect size of 0.2 corresponded to a weak-to-moderate gene effect. When comparing patients to controls, the allele powers for PCV and nAMD were 97.00% and 92.12%, respectively, and the genotype powers for PCV and nAMD were 94.01% and 86.42%, respectively.

## Discussion

Both PCV and nAMD are most common causes of irreversible visual impairment in older people worldwide. The exact pathogenesis remains unknown, and the complex genetic backgrounds remain under exploration. In this study, we first performed rs6982567 near *GDF6* genotyping in Chinese PCV and nAMD patients. The results suggested that a weak association might exist between rs6982567 near *GDF6* and PCV. The marginal association under a recessive model in the PCV and nAMD combined group might be attributed to that between this SNP and the PCV group.

*GDF6* belongs to the largest gene group within the TGF superfamily [20], which plays an important role in vascular angiogenesis and remodelling [21, 22]. *GDF6* has functions in eye and retinal development [23]. Sequence alternations in *GDF6,* such as amino acid substitutions, have been identified in some patients with ocular anomalies (microphthalmia, anophthalmia, coloboma) and vertebral segmentation anomalies [15, 24–26]. *GDF6* also participates in tissue healing and remodelling [23, 27]. Wolfman N M found that, in rats, three members of TGF-β family, GDFs 5, 6, and 7, could induce dense connective tissue formation rich in collagen type I when implanted ectopically or intramuscularly [28]. In vitro, the *GDF6* gene plays a potential role in tendon matrix modelling and remodelling [29, 30].

Bruch’s membrane is the innermost layer of the choroid and is rich in elastin and collagen. Typical findings of PCV on ICGA include an abnormal choroidal vascular network surrounded by characteristic polyp-like dilations at the border [31]. Notable sclerotic changes in PCV lesions and an elastic layer within the walls in polypoidal vessel disruption have been found in histopathologic studies [32, 33]. A mouse model of PCV demonstrated severe degradation of the tunica media of choroidal vessel walls and elastic laminae [34]. Our previous study had found that circulating gelatinase levels were elevated in PCV patients but not in nAMD patients, providing evidence of a role of extracellular matrix metabolism in the pathogenesis of PCV [35]. Considering *GDF6*’s role in retinal development and tendon tissue function, mutations of *GDF6* could result in dysfunction of vascular and supporting tissues, thus participating in the pathogenesis of PCV.

A previous study in Caucasians showed that the rs6982567 variant was associated with AMD [16]. There were two primary reasons for the inconsistency. First, the inclusion criteria were not the same. In the Caucasian study, the patients were classified according to the Age-Related Eye Disease Study classification, which included the geographic atrophy and did not specify the diagnosis of PCV. In our study, ICGA was used to distinguish PCV from AMD. Geographic atrophy was excluded from our study. We only included neovascular AMD patients. For this reason, the types of patients were not exactly the same. Second, the study populations came from different ethnic group. The same SNP might have different distributions in different populations. The *serpin peptidase inhibitor, clade G, member 1, (SERPING1)* gene was reported to be associated with Caucasian populations [36], but the results were negative in Han Chinese and Japanese populations [17, 37]. In the HapMap project, the allele frequencies of rs6982567 were 0.168 in Utah residents with Northern and Western European ancestry, 0.279 in Han Chinese people and 0.218 in Japanese people, exhibiting differences across ethnicities. These differences could constitute another explanation for the different outcomes.

There were also some limitations of our study. First, we conducted it in a relatively small sample, so we could not fully identify the associations between rs6982567 and PCV. Second, we only investigated a single SNP near the *GDF6* gene in this study. So far, other SNPs in *GDF6* locus and linkage disequilibrium (LD) analysis with this gene have yet not been completely investigated. Therefore, a complete survey of the whole target SNP in LD with the *GDF6* gene with a larger sample size would be needed.

## Conclusions

In conclusion, we investigated the associations of SNP rs6982567 with PCV, nAMD in a Han Chinese population. There was a marginal association between rs6982567 and PCV under a recessive model. Expanding the sample size and exploring the whole target SNPs in or near *GDF6* gene are needed in future studies.
